# Scapholunate tears: an update

**DOI:** 10.1186/1753-6561-9-S3-A30

**Published:** 2015-05-19

**Authors:** Christophe Mathoulin

**Affiliations:** 1 Institut de la Main, 75016, Paris, France

## Introduction

Tears of the scapholunate ligament (knowing the natural history), are difficult to treat in the chronic stage. Wrist arthroscopy has completely changed the understanding and treatment of these lesions with excellent results.

## Materials and methods

We reviewed 147 patients (91 males, 56 females) with an average age of 37.8 years (17 to 63). In 116 cases it was a sports injury, including 41 high-level athletes. The average time between the injury and presentation was 7.41 months (between 3 and 24). All patients were operated on an outpatient surgery basis under local anesthesia. After reduction of the scapholunate junction (if deemed necessary), a capsular ligament suture was performed arthroscopically using the radiocarpal and midcarpal portals. A more or less large-scaphoid bone scapholunate pinning could be combined in some stage 4 cases only.

## Results

Our average follow-up was 38.54 months (between 12 and 71 months). The pain disappeared in the majority of cases (93%), remaining just moderate in 7% of cases. We had 5 failures, all in stage 5 cases. Mobility was normal in 131 cases (82%) and with normal pronation and supination in all cases. Muscle strength improved in all cases but was below normal (as compared to the opposite side) in 18 cases.

## Discussion

Recent work in the literature showed the significance of connections between the dorsals capholunate ligament and the dorsal capsule in maintaining the stability of the scapholunate joint. In addition, also demonstrated was the importance of proprioception through intrinsic and extrinsic innervation of the scapholunate complex. Performing an arthroscopic dorsal capsuloligamentous suture provides a solution to these issues avoiding procedures resulting in stiffness that is usually seen in these cases. The quality of our repair has enabled athletes to maintain their competitive levels and at times demonstrate excellent results in international competitions.

